# Exploring students’ mathematical discussions in a multi-level hybrid learning environment

**DOI:** 10.1007/s11858-022-01364-4

**Published:** 2022-05-05

**Authors:** Chiara Giberti, Ferdinando Arzarello, Giorgio Bolondi, Heidrun Demo

**Affiliations:** 1 University of Bergamo, Bergamo, Italy; 2 University of Torino, Turin, Italy; 3 Free University of Bozen, Bolzano, Italy

**Keywords:** Complementary approach, Complexity, Digital learning environment, Mathematical discussion

## Abstract

The research described in this paper focused on the issue of describing and understanding how mathematical discussion develops in a hybrid learning environment, and how students participate in it. The experimental plan involved several classes working in parallel, with pupils and teachers interacting both in their real classrooms and in a digital environment with other pupils and teachers. The research was based on a rich set of data collected from the M@t.abel 2020 project, which was developed in Italy during the Covid health crisis. Based on Complementary Accounts Methodology, the data analysis presented in this paper involved specialists from the fields of mathematics education and inclusive education. In the study we considered the complexity of learning and the different elements that have an impact on students’ activity and participation, when they are engaged in mathematical discussions within the multilevel-digital environment that emerged due to the pandemic. These parallel analyses showed that ‘mathematical discussion in the classroom’ is a complex (and sometimes chaotic) phenomenon wherein different factors interweave. A complementary approach assists in developing a global vision for this dynamic phenomenon and in highlighting local episodes that are crucial in this interplay of factors. It is precisely in these episodes that the role of the teacher is fundamental: these episodes appear as catalysts for the different variables, with the teacher acting as mediator.

## Introduction

The Covid-19 crisis has highlighted strengths and weaknesses of education systems. The need to activate distance-learning (DL) solutions has led to enforced digitalisation which, in many cases in Italy, merely constituted an attempt to substitute the real-life classroom by transferring some elements of normal teaching practice into digital environments of communication (Bolondi, 2020). However, in Italy, the M@t.abel 2020 project, involving over 1200 teachers, was set up to develop materials and methodologies for a future scenario of hybrid teaching (Singh et al., 2021), with a multi-level model designed to foster interaction between different classes and teachers. In a hybrid environment, online components are substituted for part of the face-to-face class time while online interactions can be completed either synchronously, using real time meeting sessions, or asynchronously (Singh et al., 2021). One key objective with deep theoretical roots (for the project and for mathematics education in general) is to enhance mathematical discussion (Bartolini Bussi, 1996; Pirie & Schwarzenberger, 1988) in a hybrid environment which is technologically assisted (Sinclair, 2005), with special attention paid to the participation of all students in the discourse (Hunter & Hunter, 2018; Imm & Stylianou, 2012). Indeed, one of the main weaknesses of the educational system that became particularly visible during and after the school lock-down was its limited impact in actively addressing inequalities (Ianes & Bellacicco, 2020).

The M@t.abel 2020 project is based on the materials of a previous national project called M@t.abel, whose materials have been used by thousands of teachers and validated by numerous experiences (Pennisi et al., 2015; Taranto & Arzarello, 2020). At the beginning of the lockdown period in March 2020, the EdTech Centre of Future Education in Modena decided to adapt some of the M@t.abel materials to make them suitable for DL activities with students, thus launching the M@t.abel 2020 project (Giberti, 2022). This research was based on data collected during that project, and our aim in this paper is to provide a picture of how mathematical discussion can develop when pupils and teachers interact both in their real classrooms and in digital environments with other pupils and teachers.

Regarding methodology, the key point is that a digital learning environment allows the gathering of a huge amount of data, channelled in different forms and suitable for analysis with different techniques, both qualitative and quantitative. The Complementary Accounts Methodology, developed by David Clarke (Chan & Clarke, 2017; Clarke, 1997) and driven by different theoretical and epistemological perspectives, has been used in order to obtain the maximum possible amount of information from these data, and to focus on the connections between the information gathered via two perspectives, namely, mathematics education and inclusive education. In particular, we investigated the variables which are relevant to the description and interpretation of mathematical discussions, and the tools that may foster mathematical discussions in a Multilevel Hybrid Environment (MHE). Our research hypothesis was that a mathematical discussion based on an MHE can be useful in order to negotiate the meaning of mathematical objects and for the development of argumentation skills in mathematics, thereby facilitating the inclusion of all students.

## Research questions

The general issue we addressed was to identify the main variables useful to describe a mathematical discussion in an MHE, their role and their impact: finding these will help both teachers and researchers. This issue became crucial during the COVID-19 emergency. Distance learning highlighted this problem and at the same time made its investigation possible.

Our research questions were as follows:

1. How does mathematical discussion develop in an MHE?
2. Which aspects and features are relevant in order to describe and interpret students’ participation in mathematical discussions within an MHE, and how does this environment trigger and support such a process?
3. What can we learn from the analyses of mathematical discussions from the perspectives of both mathematics education and inclusion?

The theoretical backgrounds of the perspectives we adopted in our approach are presented in Sect. 4. The analyses and results inherent in RQ1 and RQ2 are reported respectively in Sects. 6.1 and 6.2, while complementary results emerging from the integration of the two perspectives are reported in Sect. 7.

## Literature survey

The issue of how to use digital technology in mathematics education has been studied in depth in the last few decades, and it developed in different directions thanks to the efforts of many teachers and researchers in mathematics education (Borba, 2021). One of the key elements was the emergence in the late 1990s of the Internet and online education; then, the arrival of fast Internet in many developed countries allowed new possibilities for online courses and the development of different forms of hybrid learning, i.e., integration of face-to-face and online mathematics education (Borba, 2021). Notwithstanding the importance attributed to this theme in recent years and several important research studies on implementation of online education (e.g. Engelbrecht et al., 2020b) the COVID-19 emergency highlighted a lack of research into online education for children (Borba, 2021).

In Italy, as in many other countries, the reorganisation of teaching methods was entrusted almost entirely to teachers, and teachers and learners were frequently not sufficiently prepared to tackle this technological transition (Albano et al., 2021; Engelbrecht et al., 2020a). For this reason, especially for young students, the great risk of this sudden transition to DL consisted in the loss of important pedagogical strategies based on a constructivist approach, in favour of activities based on technology but following a transmissive approach to mathematics teaching and learning (Bakker & Wagner, 2020). Furthermore, many schools struggled to stay in contact and involve students from underprivileged backgrounds and students with special education needs in meaningful learning environments (Ianes & Bellacicco, 2020). On the other hand, teachers and researchers had the possibility of learning from this difficult moment and reflecting on different aspects of mathematics education by considering *what* mathematics is needed by students, *how* to overcome a traditional way of teaching based on the transmission of knowledge also by using new technologies, and *where* to teach mathematics; indeed, the emergency period altered the boundaries between school and out-of-school contexts (Zhao, 2020). The role of researchers in accompanying teachers through this transition has been crucial during the emergency and is important today in order to “filter through all the new ideas and help select those approaches that are not only teaching students but also enabling them to learn” (Engelbrecht et al., 2020a, p. 824).

The need to propose a constructivist approach to mathematics teaching and learning, also in the hybrid environment, necessarily brings both teachers and researchers to reflect on the way that teachers and students can interact during mathematical activities, which digital tools may help develop a fruitful mathematical discussion, and how these can facilitate all students’ participation in the pursuit of inclusive education. Since the foundational paper of Pirie & Schwarzenberger (1988), ‘mathematical discussion in the classroom’ has been considered a key issue in the learning of mathematics from a constructivist approach. By definition, it is a “purposeful talk on a mathematical subject in which there are genuine pupil contributions and interactions” (Pirie & Schwarzenberger, 1988, p. 461). Thus, it includes multiple aspects, namely, those that are mathematical of course, but also social, emotional, and cognitive aspects. It takes place in a concrete environment (a classroom, a platform) and relies on a variety of mediating agents and instruments active therein, including the following: the teacher, the class group as a whole, the cultural instruments of mediation (language(s) used in the classroom, chosen semiotic resources), and different technical tools (paper and pencil, blackboard, whiteboard, computers, apps). Since then, a large amount of research has been carried out on the different facets of mediation in the classroom, according to different theoretical perspectives, including Vygotskian (Wertsch, 2007), semiotic (Presmeg et al., 2016), and instrumental (Trouche & Drijvers, 2014). The complexity of mathematical discussion as a multifaceted activity and the role of the teacher in this has since been specifically highlighted (Bartolini Bussi, 1996; Cirillo & Herbel-Eisenmann, 2009; Walshaw & Anthony, 2008).

Furthermore, the discussion analysed in this paper is based on a comparison of alternative solutions to the same problem: as already highlighted in previous literature, this is a powerful practice for reaching conceptual understanding of mathematical concepts and developing relational thinking in mathematics (Richland et al., 2017). One important feature of an MHE is that the discussion in the classroom may develop through a network of synchronous linking between statements, replies, and comments posted (for instance, in a Padlet), and this adds a new dimension to the above-mentioned complexity: in a ‘traditional’ classroom discussion, the discourse develops mainly along a linear timeline.

## Theoretical perspectives

In this section, we present the relevant main specific references of the different perspectives. In view of a complementary approach, different ‘researchers’ eyes’ were used in order to highlight key points of the mathematical discussion. The interplay of these perspectives is explored in Sect. 8, where all ‘eyes’ focus on the same episodes.

### Mathematics education

The mathematical analysis is based on the following complementary frameworks:

- The *focal* and *preoccupational* analyses by Sfard (in Kieran et al., 2003, pp. 34–44) to analyse students’ and teacher’s interactions: the former pictures their conversational flows and the latter considers also their meta-messages and engagement while interacting with each other;
- The verbal and gestural semiotic resources produced in the interactions, respectively scrutinized through the *textual analysis* by Scholes (1985) and the *gesture classification* by McNeill (2005);
- An extended version of the Webbing and Instrumental Orchestration approach by Trouche and Drijvers (2014). This perspective allows us to expand the analysis of specific and complex forms of interactions within the MHE of our teaching experiments.

Each of these theoretical frameworks allows us to focus on a specific facet of the mathematical discussion. Students’ and teacher’s interactions can thus be classified according to the following lenses:

- *the (discursive) focus*, the expression used by an interlocutor to identify the object of his/her attention, which has three main elements (Kieran et al., 2003, p. 34):
  - *pronounced focus*: pronounced words;
  - *attended focus*: scanning procedure with which the subject accompanies the words, to address what she/he is referring to; and.
  - *intended focus*: this relates the speaking person to an assortment of statements he or she is now able to make regarding the entity identified by the pronounced focus;
- *the flow of interactions*, which reveals the interest of interlocutors in creating a real dialogue with their partners and offers three types of interactions (Kieran et al., 2003, p. 41):
  - *reactive*;
  - *proactive*;
  - *both reactive and proactive*.
- *the semiotic resources produced by the participants*, which can be as follows:
  - *verbal production*: produced by reading what is written and classified (Scholes, 1985, p. 24) as follows:
    - *text within text*: when reading;
    - *text upon text*: when interpreting;
    - *text against text*: when criticizing.
  - *gesture*s: synchronous with verbal productions classified as follows (McNeill, 2005, p. 39 ff.):
    - *iconic*: reproducing the physical form of some object;
    - *deictic*: indicating something which is present;
    - *metaphoric*: referring to some abstract concept;
    - *emblematic*: sharing a cultural social meaning.

A theoretical composition and integration of these tools is compacted in the infographics reported in the following sections, revealing the complexity of the structure and its dynamic evolution. As discussed in Sect. 6.1, the resulting structure of the discussion will show that the specific affordances allowed and promoted by the MHE produce an extended picture of the Webbing and Instrumental Orchestration described by Trouche and Drijvers (2014).

### Inclusion

The perspective of inclusive education focuses on each student’s presence, learning and participation within learning situations and settings (Ainscow, 2016; Slee, 2018), with the general aim of contributing to the development of equitable learning environments (IBE-UNESCO, 2016). Students are not investigated as a homogeneous group; instead, their individual and unique ways of learning and participating is the focus.

In general terms, participation defines an expected effect of inclusive learning settings and implies subjective social well-being and the opportunity to actively contribute to interactions taking place in schools (Booth & Ainscow, 2002). The construct is defined differently according to different levels of the school system (macro level of the national system, meso level of the single school, micro level of the class) (Ianes, 2021). For the purpose of this paper, reports of individual student participation in the whole class discussion are presented. Methodological elements from Conversation Analysis that describe what social actors say and do in interactions were adopted, as they had already revealed themselves particularly useful for the study of participation at the micro-level of classroom interaction (Demo & Veronesi, 2019). From this perspective, in the data analysis of the final discussion by class A, taking part in the interaction is interpreted as visible evidence of participation.

Secondly, an interesting contribution to the understanding of participation in teaching and learning has been offered by research into cooperative learning, which has studied students’ social competences that make equal participation in joint collaborative learning situations possible (Cohen & Lotan, 2014; Comoglio & Cardoso, 1996). Scholars have described how participation in cooperative learning situations requires a variety of social competences that can be organised on a scale of increasing complexity. Being present, and watching interactions take place, can be considered the initial stage of the scale that gains in complexity as the student also answers or reacts to teachers’ or classmates’ communication initiatives. At the highest end of the scale, we find social competences related to leadership. Students display this competence when they lead the joint discussion in a certain thematic direction, putting forward new or different topics for the conversation and exposing themselves to the risk of errors that may become visible to other group members or the teacher. This organisation of social competences allows the description of individual students’ participation in a more accurate way, referring to the level of social competences they are activating in single episodes. Against this background, the teacher’s reactions to students’ divergent interventions in the final discussion by class A were analysed and interpreted in terms of support given to student participation.

The integration of technologies in inclusive classroom environments has been broadly discussed within literature and research involving Universal Design for Learning—UDL (Rose & Meyer, 2006). The UDL approach puts forward three principles for making the learning environment accessible to everyone *a priori* using the strategy of pluralisation, promoting the following: (1) plural representation of new knowledge, for example both via verbal and visual means; (2) plural opportunities of expression, for example letting the students choose between visual or written presentation of their work; and (3) considering the students’ different interests and preferences in order to sustain their engagement in learning. Technology plays a crucial role in this endeavour because it facilitates the enrichment of the environment. On the other hand, plurality is not enough: more means do not linearly increase accessibility and participation. A coherent coordination of the different means is necessary, with a careful reflection on the risk that plurality of means becomes chaotic and, in that way, paradoxically an obstacle for some (Mangiatordi, 2022).

## Methodology

In Clarke’s approach (1997), “Complementary accounts methodology is distinguished […] by (1) the nature of the data collection procedures, leading to the construction of ‘integrated data sets’ […]; (2) the inclusion of the reflective voice of participant students […]; (3) an analytical approach that utilizes a research team with complementary but diverse areas of expertise” (p. 98).

Our digital teaching/learning environment allowed immense data-gathering, channelled in different forms and suitable for analysis with different techniques, both qualitative and quantitative. Our data set was constructed by integrating data from the data sources of technologically-mediated discussions and face-to-face classroom interactions. The expertise areas of our research team integrated different but complementary perspectives, namely, those mathematics education and inclusive education. Our methodology, based on the Complementary Accounts Approach, worked as follows:

1. an experiment plan was designed to generate and collect structured data from multiple sources;
  - ‘rough’ data were collected both from classroom-videos and through the platform-Padlet discussions;
  - these data were organised in exports of the Padlet discussions and transcripts (with numbered lines and description of gestures) of the recordings;
2. students’ reflective voices were extrapolated from the discussion and teachers’ voices were collected from focus groups;
3. researchers from the fields of inclusive education and mathematics education were involved, who contributed the following:
  - they concurrently analysed the same data, explicitly defining the meaning they gave to keywords of the research questions from their own theoretical perspectives, highlighting key points emerging from the analysis, and identifying crucial episodes;
  - they initiated a common discussion to define episodes revealed as significant from all perspectives.

Indeed, this methodological plan included different focus groups involving teachers and researchers who collected and shared information about the activity, the classes involved and who reflected on the outcome of the experiment. Other focus groups involved only researchers, who shared the meanings allocated to specific terms used in different disciplines (e.g., ‘discussion’) and compared the results from different perspectives, allowing the identification of those results reported in Sect. 7.

In this paper, we do not consider data from the teachers’ focus group, but concentrate mainly on the mathematical discussion of class A; the discussion analysis was performed by the researchers and not by the teachers. We report here the parallel analysis, in which each researcher adopted the specific analytical tool of his/her framework, and the common discussion of one episode, which occurred during the final discussion in class A (see below). The transcript of this episode is in the Appendix.

### Experiment plan and data generation

#### The task-related stimulus for the discussion

**Fig. 1:**
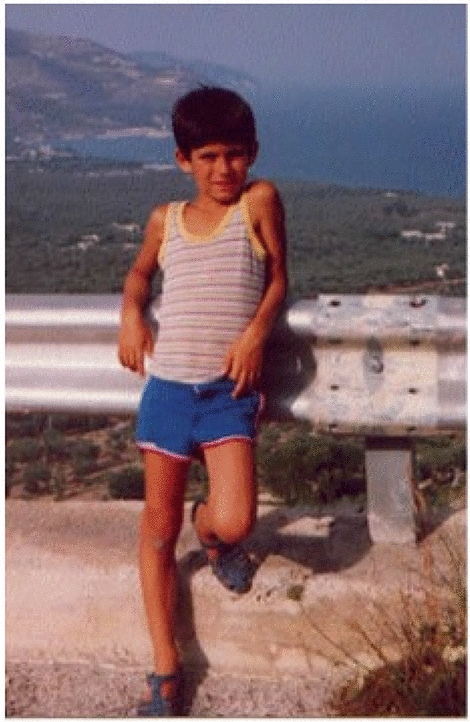
Picture focus of the M@t.abel activity

We used the problem situation of the M@t.abel activity ‘La Foto’ (The Picture).^1^ In this activity students were asked to calculate the height of a child in a picture (Fig. 1). This is a typical problem-solving situation: learners do not have any routine procedure to identify the solution and many possible strategies might be adopted. For instance, they could reason on relationships between the dimensions of objects in the picture, but they could also base their assumptions on data driven by other sources (web, personal experience, etc.). In this activity, the way the teacher presents the problem to students is extremely important: the goal must be clear and students need time to reflect individually on possible strategies, assumptions and solutions. Then, all students’ ideas must be shared and discussed within the class.

In our study, we used a new version of the activity, developed as part of the M@t.abel 2020 project and already tested in several classes. In this new version, students and teachers could participate in the discussion by posting texts, links, videos, images, and files and commenting on each other’s ideas on an online notice board such as Padlet (our selected platform). The task was proposed by the teacher during a lesson. Furthermore, the original picture was included in the Padlet with the request in written form. Then students had to work individually: each student posted her/his own solution on the Padlet without the possibility of seeing their classmates’ posts; this gave them the chance to take time to think and explain their ideas. When all the students had posted their solutions, the teacher approved the posts and made them visible to everybody. In this way, it was possible for everyone to read and comment on the posts of any other classmate, while allowing the teacher to keep a written record of their comments. The digital setting of the activity implemented on a platform allows students and teachers to share Padlets (and hence interlace discussions) also with other classes and students, while researchers can collect quantities of data beyond the possibilities offered by separate classroom observations. Thus, the mathematical discussion starts from this online notice board, which becomes the stimulus for a final mathematical discussion in the classroom.

#### Participants

The experimental plan involved two sixth grade classes (class A composed of 25 students, and class B composed of 18 students) from two different secondary schools in Italy. The two classes were from the same province (Bologna) but from different areas and backgrounds: class A was from a small school in the mountains near the city while class B was from the city centre. Considering the socio-economic background of families, and students with immigrant backgrounds, the background of class A was ‘lower’ than that of class B; for instance, the percentage of students with an immigrant background was higher in class A even though almost all of these students were second-generation immigrants with no special needs arising from language difficulties. In both classes, two students were identified as students with special needs due to specific learning difficulties, but no student with certified disability was present. The students of the two classes did not know each other: this allowed them to comment more freely on the Padlet of the other class, without any possible conditioning; the two classes met each other only after the research period ended. The teachers involved in the research did not know each other, meeting only in the focus group session, from which moment on they had the opportunity to collaborate in planning the timeline of different phases of the research.

### The teaching experiment

The teachers of the two classes proposed the stimulus activity in each class separately, collecting students’ ideas in two different Padlets (Padlet A and Padlet B). When all the students had posted their solutions, the teacher approved the posts and made them visible to the other students in the class. Concurrently, to bring the experiment to a multiclass level, the teacher sent a copy of the Padlet to the teacher of the other class. Students thus had the opportunity to read and comment on the posts of their classmates and, at a later moment, were encouraged to read and comment also on posts by the other class. The final discussion was thus based not only on their own class Padlet but also on the Padlet shared by the parallel class. Students did not use their real names in the Padlets, to guarantee privacy and confidentiality; they used nicknames which were shared only in their class and were used also during the final discussion.

Data analysed in this research pertained only to class A and its multilevel discussion itinerary, which also involved class B. Data regarding class B exclusively were not considered in this work, in order to focus all the analysis from different perspectives on the same discussion. Hence, all the researchers analysed the following data:

- Student Padlet responses of Class A, commented on by students from Class A;
- Student Padlet responses of Class A, commented on by students from Class B;
- Final discussion by class A (video and transcript).

## Parallel analyses

According to our experimental plan, students posted their solution on a Padlet and then the discussion was launched via the students’ comments before being developed further during the lesson referring to the Padlet posts and comments. For this reason, we could consider some of the variables describing a mathematical discussion identified by Pirie and Schwarzenberger (1988) as fixed:

- the *actors* were the students of the two classes involved, the teacher, and the class group as a whole during the discussion;
- the *environment* included not only the classroom but also the platform, which represents the technical modality with which students had to post their solutions (written posts, pictures, audio) and comments (written posts).

Other variables, for instance the cultural instruments, might be influenced by the use of the Padlet and depend also on the way the teacher conducted the discussion; moreover, they might be influenced by comments from the other class.

**Fig. 2:**
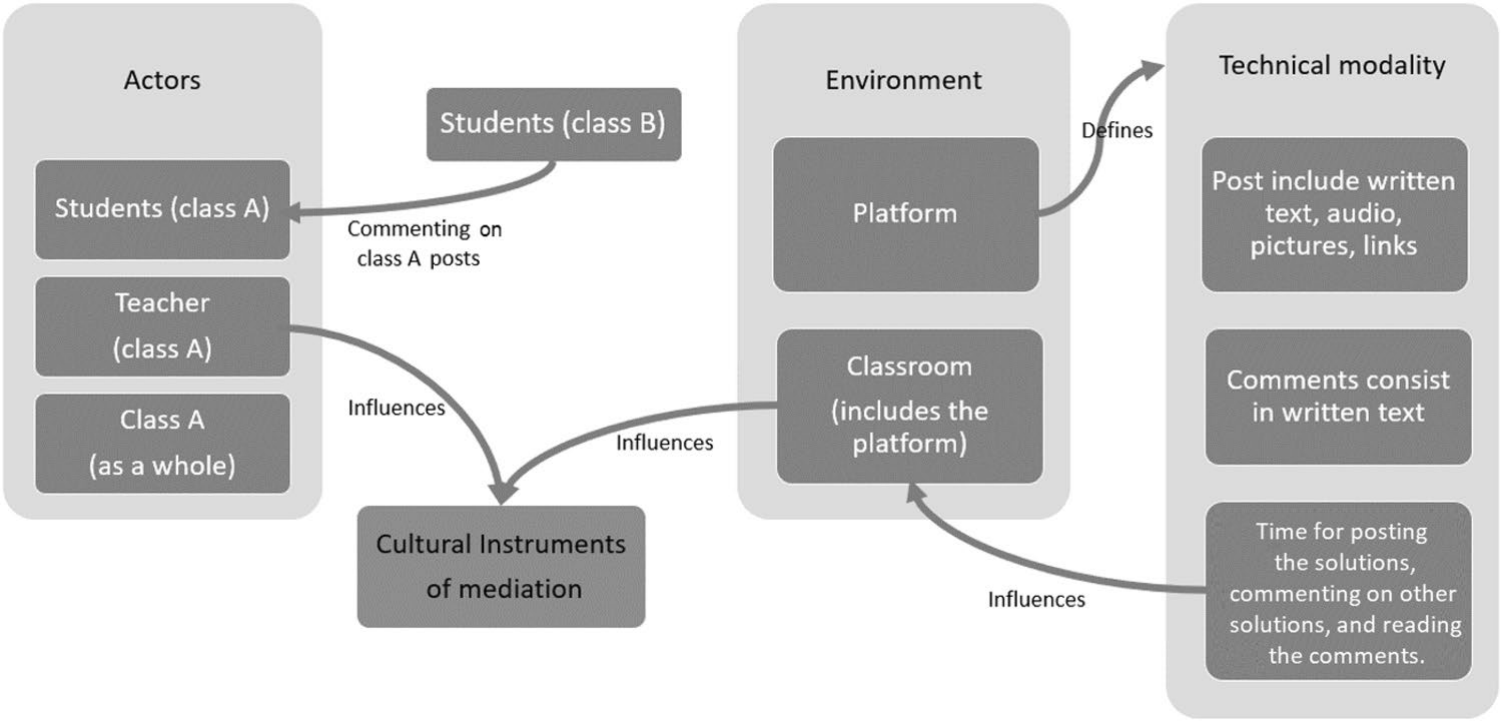
The dynamics of the discussion in the MHE

Our main focus is on the introduction of the MHE in which the discussion takes place, and in the new offerings that it provides for investigation (Fig. 2).

In this section we report the results of the parallel analysis of one specific set of data included in the appendix, taken from the final discussion in class A, and in particular of one episode (lines 40–140 of the transcript), which was highlighted as important from the two perspectives. We chose this episode since both hints and stimuli from previous passages of the discussion re-emerged, and this episode itself re-emerged in later passages. Moreover, the crucial words ‘high/height’ become here the focus of a classroom discussion stimulated by the analysis of two Padlets (Padlet A commented on by Classes A and B; Padlet B commented on by Class A). The results of the analysis according to these two perspectives (mathematics education and inclusion) are particularly interesting and highlight different aspects of the discussion.

### Mathematics education

In this section, we use the specific components of each of our theoretical constructs to analyse the final mathematical discussion of class A, based on the texts posted in the Padlets. Together, they contribute to offering a complete and more compact picture of the meaning of the classroom interactions from a mathematical standpoint.

First, the presence of the Padlet enlarges the configuration of the classroom environment described by Trouche and Drijvers (2014), increasing the number of *discussion levels of interaction* to four:

- the solutions students posted on the Padlet (A);
- their comments on the posts they uploaded regarding their solutions (A→A);
- the posts that other class students uploaded to comment on solutions in A (B→A);
- f2f interactions between the teacher and students in the classroom (C): these may possibly concern the content of each of the previous levels, which is shown via the use of the whiteboard in the classroom, or discussions, which gradually developed in the classroom.

**Fig. 3:**
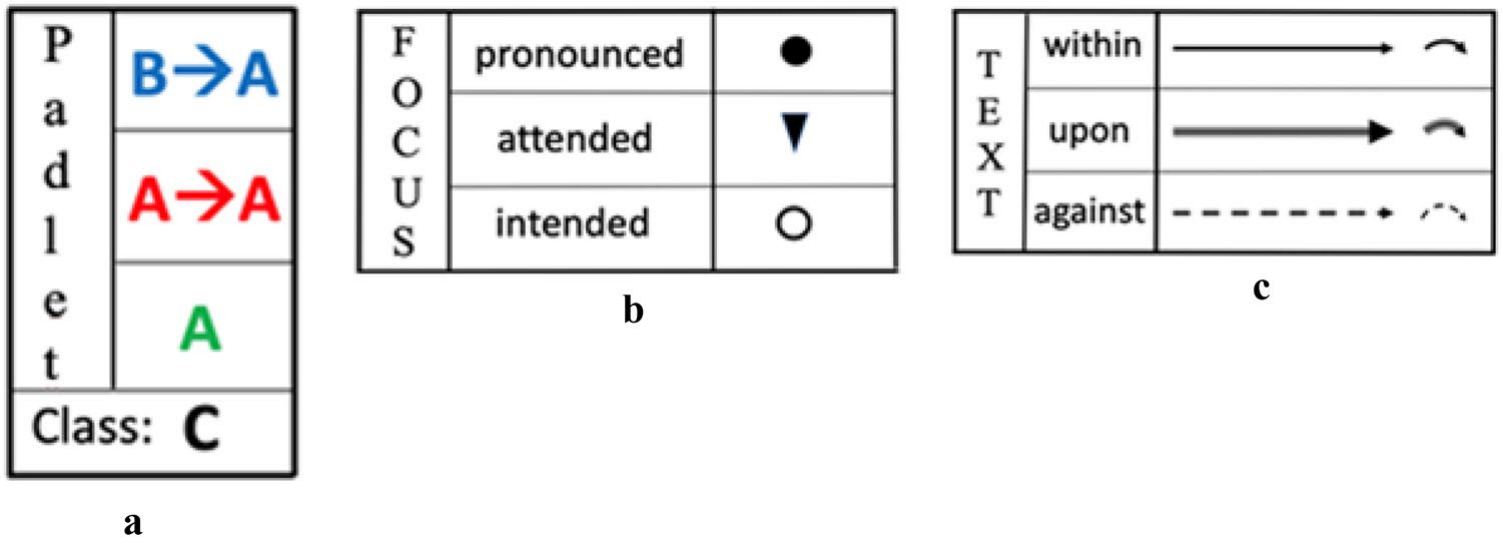
Graphical representations used in infographics

We use the graphical representations pictured in Fig. 3 to represent some of the different lenses of our analysis described in Sect. 4.1. In Fig. 3a, colours indicate the different *levels of interaction.* Figure 3b shows the graphical representations of different types of *discursive focus* and Fig. 3c represents different type of *verbal production* produced by reading the content in the Padlet. The arrows are diagonally backward when reactive, diagonally forward when proactive; when the same subject produces a contribution which is proactive but a reaction to a previous one, two arrows (one forward, the other backward) are used. Focal and textual symbols display the colours of the level of interaction within which they are produced. The small curved arrows (Fig. 3c) indicate cases in which the subject proceeds with her/his interventions, thus delving deeper into her/his discourse all the time.

**Fig. 4:**
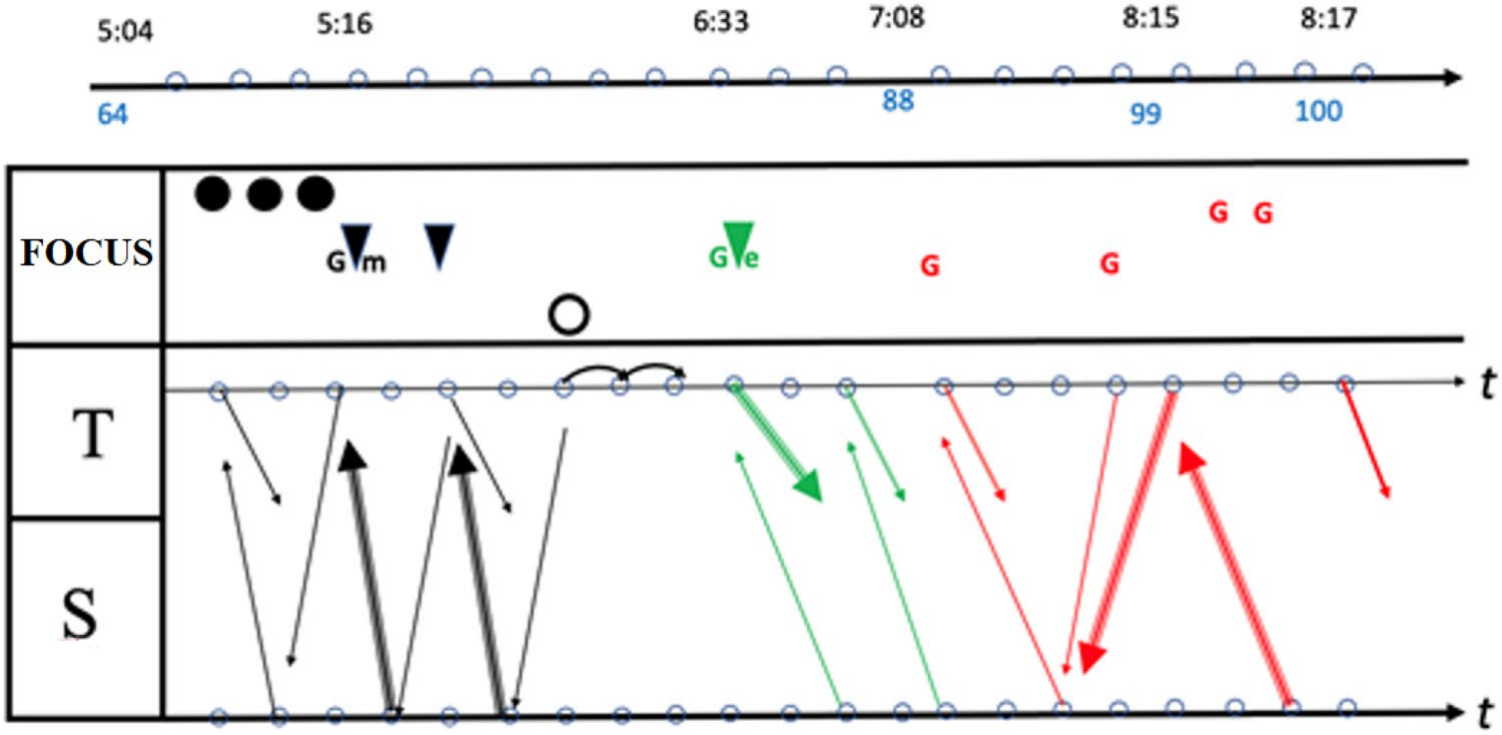
Infographic of episode from t = 5:04 to t = 8:17

**Fig. 5:**
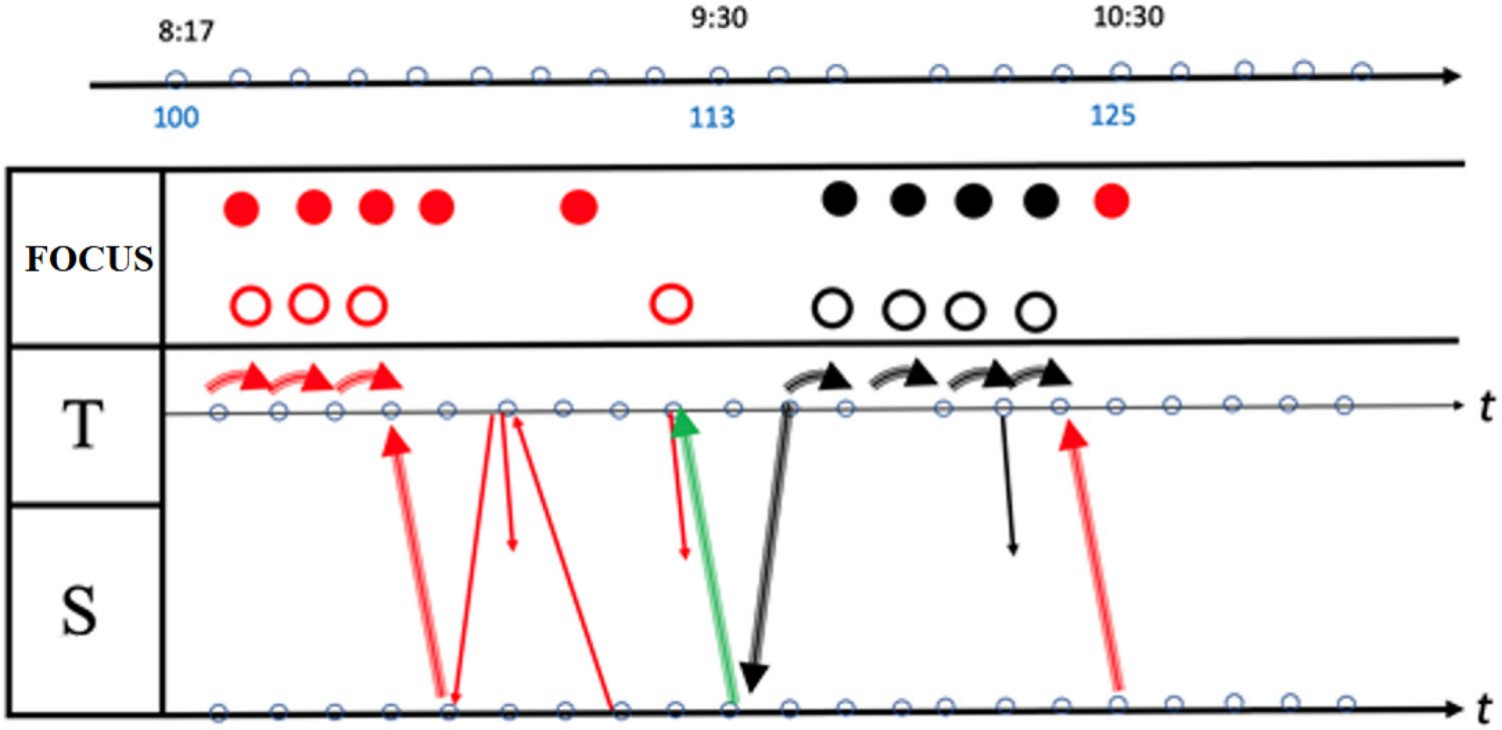
Infographic of episode from t = 8:17 to t = 10:30

When a gesture occurs, it is indicated with the letter G, followed by the initial for its specific type: for instance, Gm indicates a *metaphoric gesture*, Ge indicates an *emblematic gesture*. Where this classification is dubious, we use the letter G alone. All these symbols were collected in an infographic. Within this, a time-line was also presented: hence each symbol was drawn at a certain precise moment. The infographic created analysing this discussion, which also includes the use of the Padlet, was an extended version of infographics presented in previous work regarding other classroom discussions (Arzarello et al., 2011; Arzarello, 2017). An example of our infographic is given in Figs. 4 and 5, where the episodes of the video from t = 5:04 to t = 10:30 are represented (l.64–125).

**Fig. 6:**
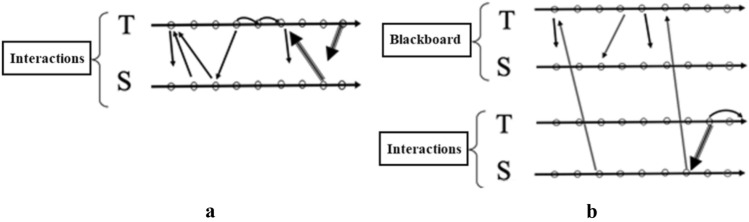
Standard interactions in classroom, verbal only (**a**) and using the blackboard (**b**)

**Fig. 7:**
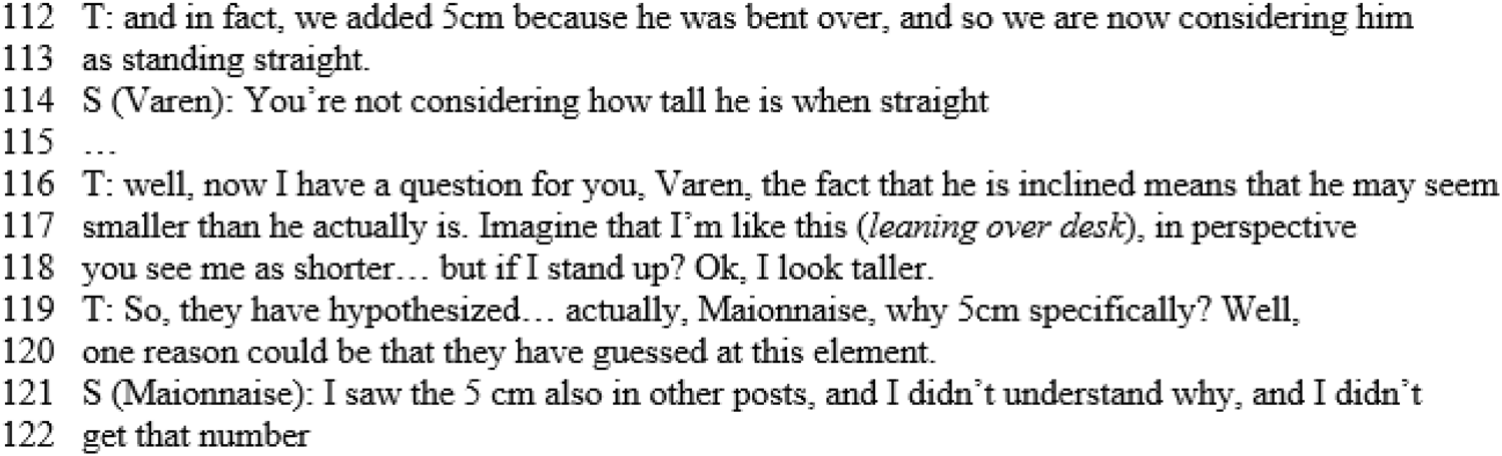
Discussion A from t = 9.25 to t = 10.30

**Fig. 8:**
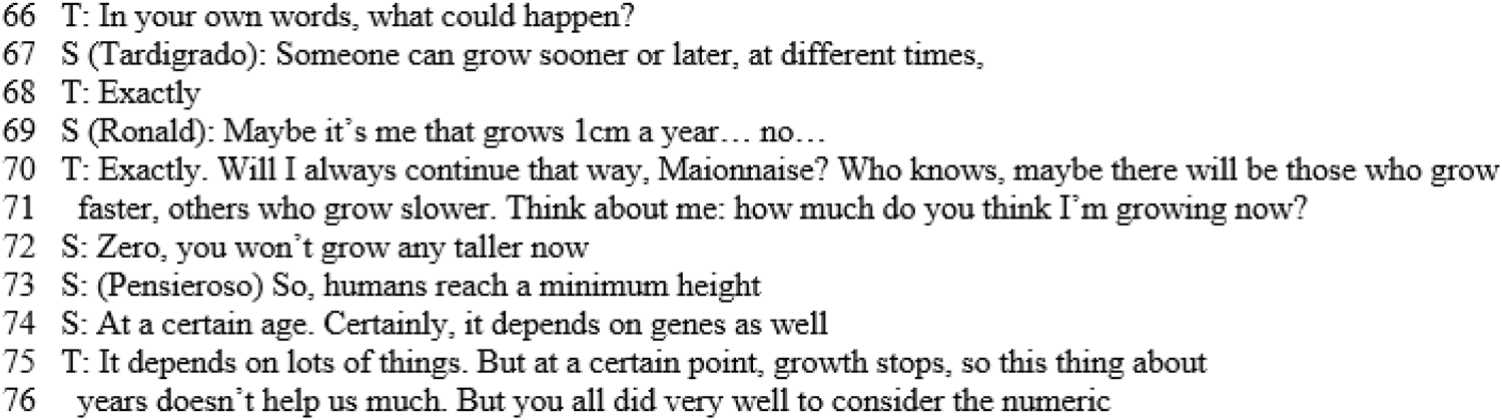
Discussion A from t = 5.08 to t = 6.03

Each infographic is divided into two bands. In the upper band, focal features are marked, with colours that show the interactive level of each one; gestures are also indicated in this zone. The lower band contains the outputs of the teacher (T) and students (S): each arrow indicates the textual aspects of the production, who is doing that, at what instant, and towards whom it is reactive or proactive. In this way, we obtained all the information necessary to draw some conclusions about the global features and dynamics of the discussion, which are outlined in the Sect. 8. The infographic shows the most significant qualitative difference from usual classroom discussions (Fig. 6a and b). The different levels introduce an effective generator for the dynamics of the discussion. The Padlet puts forward a variety of voices (Bartolini Bussi, 1996; Wertsch, 1991): the teacher or students can re-voice them in a reactive/proactive form, producing texts upon or against the texts in the Padlet. Reactive form expresses the fact that the source utterance is a reaction to the target utterance: see, for example, Varen in l.114 of Fig. 7. Proactive form symbolizes the fact that the source utterance invites a response, so that the following utterance is expected to be a reaction: see, for example, the teacher’s question in l. 71 of Fig. 8. The discussion can thus be described with the metaphor of “a polyphony of articulated voices on a mathematical object”, introduced by Bartolini Bussi (1996, p. 16) and based on the ideas of Wertsch (1991). The infographic of Figs. 4 and 5 is like the score of this polyphonic production, where the teacher plays a crucial role in orchestrating it (Bartolini Bussi, 1996, p. 17).

Due to the Padlet’s role, the idea of the discussion as a polyphony of voices orchestrated by the teacher has a strong similarity with the concept of web orchestration described by Trouche and Drijvers (2014; Fig. 6), adopted to analyse discussions in an MHE. They based their model on the crucial role of the Sherpa in the discussions (pp. 196, 197), the Sherpa-student uses technology to present his/her work to the other students: in our case, the different level texts in the Padlet are used by the teacher with a ‘sherpa function’ to promote the interactions of students, and sometimes students and/or the teacher mimic what is suggested by another classmate’s Padlet text to grasp or discuss a mathematical idea (see, for example lines 60–62, or 116–118). Indeed, in the extract represented by the infographics the teacher uses the Padlet with ‘a sherpa function’, reading a post and integrating students’ voices with comments (verbal production within and upon text, Fig. 5) and gestures (from t = 8.00 to t = 9.00). In the post, the students proposed an estimation of 125 cm for Luca’s height: they considered 120 cm comparing Luca’s body with the elements in the picture, and then stated that “the body is sloping, and we have to add another 5 cm, so Luca’s height is 1.25m”. This statement promotes a reflection by another student (Maionnaise) who asks “Why 5 cm, and not 7 or even 3 cm? Why 5 exactly?” (line 105, t = 9.06, red arrow backward, reactive and upon text). This issue is then discussed in the classroom also by other students led by the teacher in a mediator role (l.105–111, following red arrows both reactive and proactive within text, Fig. 5).

This extract finished with a final contribution by the student who proposed the question, explicitly referring to the post of another student in the Padlet and his reflection on that (last red arrow in Fig. 5).

**Fig. 9:**
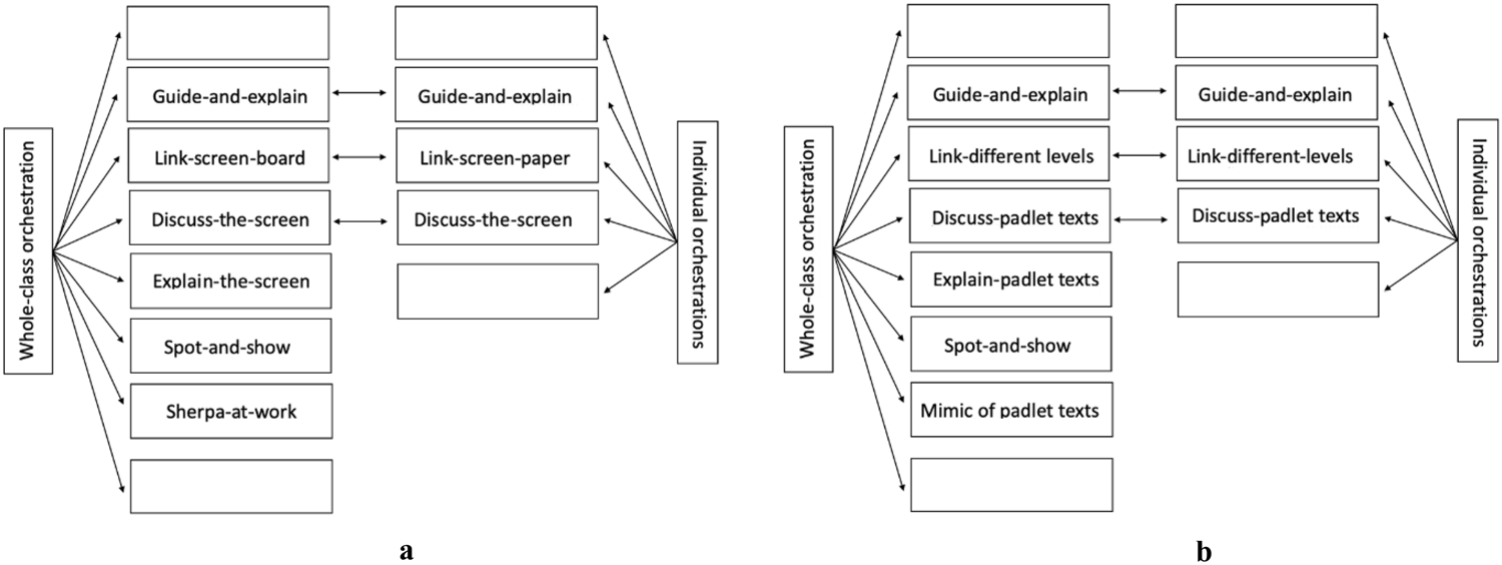
Comparison between Trouche and Drijvers (**a**) vs. Padlet (**b**) orchestration

The class-levels, focus, text, and gesture rubrics represented in our infographics feature the structure of the orchestration within the new environment. In Fig. 9a, b, we point out similarities and differences between the web orchestrations described by Trouche and Drijvers (2014, Sect. 5.1) and those in the Padlet environment. The changes are due to the functions of the Padlet: it substitutes the screen of the Sherpa in the interactions, so promoting the different levels of the discussion. The orchestration has the same structure as that described by Trouche and Drijvers, but the screen functions are substituted by the corresponding Padlet ones (see Fig. 9a, b).

**Fig. 10:**
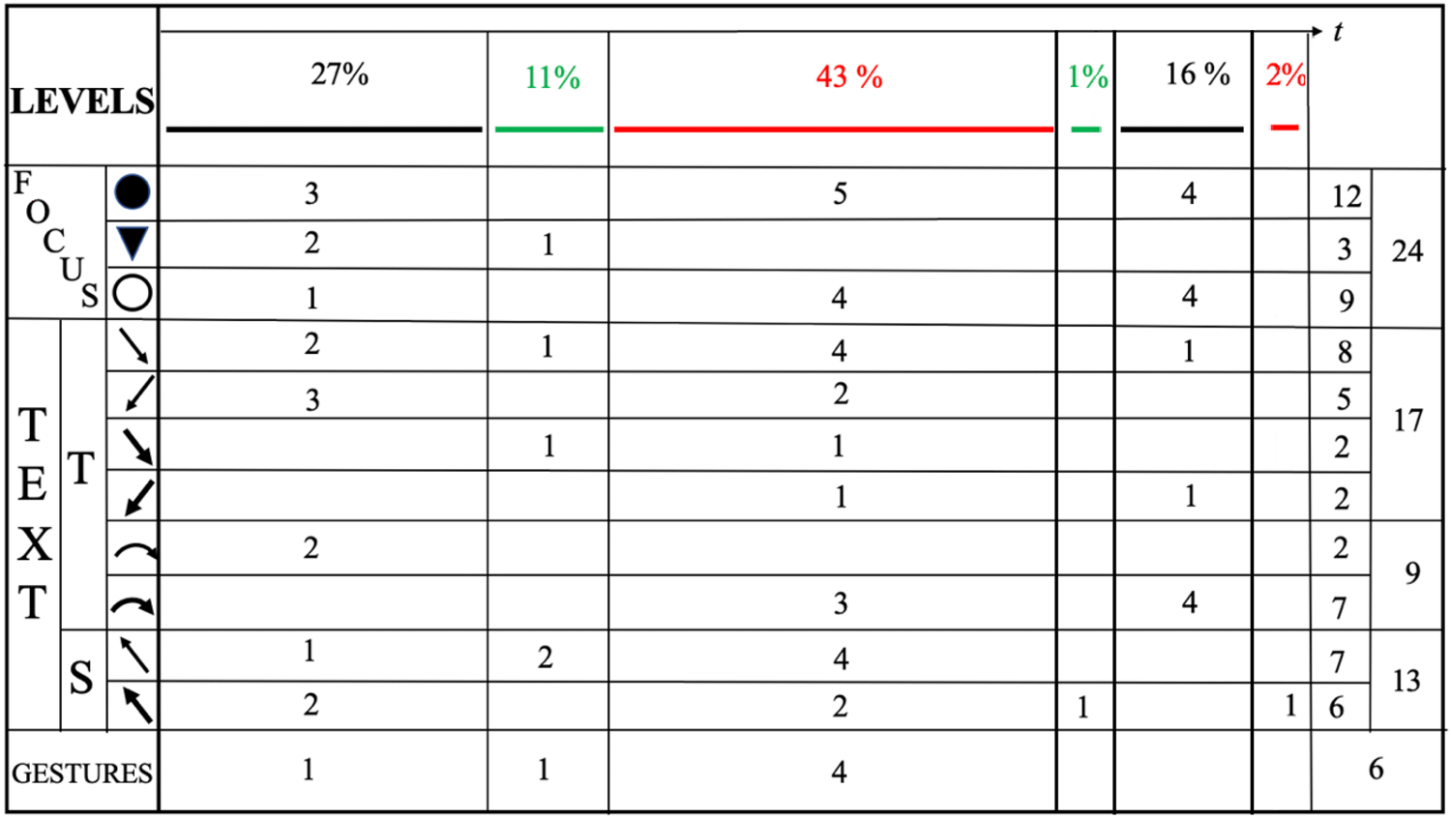
Statistics of the infographics

The statistics from the infographic of Figs. 4 and 5 supply interesting information about the global dynamics of the interactions, as shown in Fig. 10. Here we limited ourselves to collecting only some key observations, which provide the basis for the discussion.

An initial reflection concerns the percentages and distribution in time of the levels of interaction: 88% concerns the C and A→A level, with a small percentage (12%) for level A; moreover, the discussion of level A→A is almost completely concentrated in the middle of the discussion, which starts at level C and continues for a shorter time at that level after the central A→A discussion. In fact, the discussion starts with ‘external’ references to real life, which concern the height of the boy. In this first part, the level of interaction is the classroom level; indeed, the students and teacher discuss the idea that the growth of a person is not a linear process (Fig. 8) before deciding that it is not correct to divide the height of Luca by 5 and then multiply it by his actual age.

In this interaction, the focus is firstly ‘pronounced’, then ‘attended’ and finally ‘intended’; we observe the use of gestures, and verbal production both within text (reading) and upon text (interpretation). The two small curved arrows at the end of this part highlight a progression in the discourse made by the teacher but based on previous interactions.

Then, solicited by the teacher (l.90–1.91, “Let’s look, for example, at those you liked. Ok?”), it switches to a debate on comments made about the solution of the problem uploaded in the Padlet. After that, again following a question from the teacher (l.116–1.118, Fig. 7, “[…] in perspective you see me as shorter… but if I stand up?”) the discussion turns once more to ‘external’ considerations (level C), but now based on the previous discussion of level A→A. This shows the fruitful intertwining between the levels as an active promoter of interactions and of new ideas, also thanks to the interventions of the teacher, who is very proactive in this regard. It is also significant that almost all the intended foci (8 out of 9) occur in the second part of the discussion. The intended focus marks, in a sense, a more reflective engagement about the ‘why’ as opposed to simply the ‘what’ of things: in the first discussion of level C, there are 3 pronounced foci and 1 intended focus, while in the last two discussions of level A→A and C there is a balance between them, namely, 5 − 4 and 4–4 respectively. Moreover, from l.90 onward, a conflict emerges between the height of the boy and the height of his head (intended as the measurement of the distance between his head and the ground). Measurements of other elements of the picture are considered, such as those of the sides of a ‘proto-triangle’, two sides of which are the body of the boy, with the vertical drawn from his head. The teacher mimics this triangle with her body (l.119).

Another interesting point concerns the text. First of all, within this episode, the ‘against’ modality is missing: this possibly means a collaborative climate in the discussion with a joint endeavour towards shared knowledge. A second issue concerns a peculiarity of the two main sections of the discussion: all the teacher’s interventions are concentrated here, classified with a fat curved arrow; it means that the teacher is pushing the discussion about interpretation of what is under analysis. The answer of the students is more proactive in the A→A discussion than in the last C level one: we have 6 vs. 0 reactions to the suggestions of the teacher, with an interesting interpretative reaction again in the last A→A discussion.

### Inclusion

From the point of view of inclusive education, the analysis had two foci. Firstly, the relationship between the digital and the face-to-face learning environment was analysed in terms of participation, looking at the way the posts and commentary activity in Padlet facilitated students’ participation in class discussion. Secondly, the teachers’ reactions to students’ posts that propose new and divergent issues for the mathematical discussion—with a contribution in interaction that implies leadership social competences—were analysed. From the perspective of inclusive education, this is crucial in term of participation because it shows how a teacher’s feedback on students’ contributions can support or hinder the development of complex social skills that strengthen student participation.

For the first focus of analysis, the transcript of the final discussion by class A was analysed, with particular reference to the sequences where students who posted in the Padlet participated in class discussion. Participation can take different forms: providing an answer to teachers’ questions, asking a question, or simply being referred to within the teacher’s interventions (Table 1).

**Table 1:** Analysis of participation of students who posted on the Padlet to the class discussion

Ginger (G)	Teacher (T) reads aloud G’s posts (238–246)^a^
Maxinne (MA)	T reads aloud MA’s comment on other posts.
Maionese (M)	M gives answers to T’s questions in numerous sequences (25–26; 105–107; 121–122; 229; 252–256; 264; 520–525; 549) One sequence is activated by his question (121–182). At line 200, T refers to the number of likes M’s post received in the Padlet.
Artica (A)	T reads aloud A and TA’s joint post (94–104) and A’s comments on other posts (222).
Tardigrado (TA)	T reads aloud A and TA’s joint post (94–104) and refers twice to TA’s comment on another post (272; 311) TA gives answers to T’s questions in numerous sequences (331; 349; 365; 393–415; 417–422; 444–473; 497–507; 518).
Jaguar (J)	T reads aloud J’s post for the class (219–223), refers to a comment received by J (370–379) and to one made by J on another post (509–5013).
Ronald (R)	R speaks actively in numerous sequences (31; 43–88; 92; 196; 342; 374; 478–488) R asks to read and discuss his own post (303–335)
Volpe Rossa (VR)	T reads aloud VR’s post and then the teacher and classmates pose some questions about it (268–282)
Varenne (V)	The teacher introduces the group to V’s idea contained in the post (387–411). At line 108, V asks a question.
Airys (AI)	AI speaks twice (124; 249–250)

^a^ The teacher might have addressed Ginger in another situation (412–413), but it remains unclear from the video and the transcript

Summing up, of the ten boys and girls who posted a comment in Padlet, all were named in the class discussion with one exception (AI). For five pupils, no participation other than the teacher referring to them, by reading their post and/or comment, is documented in the transcript (VR, G, MA, A and J). The Padlet seemed to play an important role in facilitating participation in the contributions by students who choose not to speak in a class discussion.

As regards social competences required for active participation, the literature dedicates particular attention to the ability to propose new issues in discussion, and considers this skill to be a high-level social competence related to leadership (Comoglio & Cardoso, 1996). In fact, introducing new issues implies a higher risk of error as compared with interventions that confirm or develop topics which have already received positive feedback from the group and/or teacher. From this perspective, we analysed the way the teacher conducts a class discussion on four posts written by students who adopt divergent strategies to estimate Luca’s height in the picture (Table 2).

**Table 2:** Divergent assumptions and their discussion in class

	Content of the post	Class discussion
Blu (B)	In my opinion, Luca needs to divide his actual height by 5 and this should result in his height at the age of 5	B is not present. T corrects all assumptions that refer to “time” in order to estimate Luca’s height
V	Luca could look for his old clothes. If he puts them together, we will be able to work out the requested height.	V’s assumption is discussed and accepted as correct (387–411)
VR	The guardrail behind Luca is about 18 cm. These 18 cm are repeated in Luca around 5 times: 18 × 5 = 90 cm. Thus, Luca is about 1 m and a half high.	VR’s assumption is discussed and accepted as correct (268–282)
R	Maybe, as he is next to the barriers, if I put another one on top, that could equal his height	R asks to discuss his assumption and it is accepted as correct (303–335)

Only one of the divergent assumptions was considered incorrect in the class discussion. In this case, it is particularly interesting to analyse the way the teacher gave the corrective feedback (l.49–1.83). To correct the age-related assumption, she did not link the incorrect strategy to the name/post of a specific pupil, but encouraged the whole class to reflect on the error. To do so, she used a question to suggest a counterfactual example: “But do we grow at the same rate every year?”. The sequence was closed with an explicit correction of the initial assumption and evaluation of positive elements of the wrong answer (“This thing about years doesn’t help us much. But you were all very good to consider the numeric…”, l.75–1.76). The other three assumptions, even though divergent, were considered acceptable and correct. For each of them, the teacher made an effort (sometimes with the help of questions posed by other students) to understand the perspective of the student who generated the assumptions, and support the classmates’ understanding of it. An example of this is the teacher’s gestural attempt to make R’s unconventional proposal for a unit of measurement comprehensible to the class (l.303–1.335); R proposed using the guardrail in the picture as a unit of measurement and the teacher, indicating the picture on the board with her fingers, replicated the height of the guardrail many times to represent the height of the child.

In brief, these results on teacher feedback regarding students’ divergent assumptions suggest the teacher’s commitment to the following: (1) a positive culture around errors, which are not stigmatised as faulty but treated as productive opportunities for reflection by the whole class, and (2) the recognition of multiple and equally valid means of expression and contribution to knowledge co-construction. More in general, coming back to the focus on participation, the teachers’ welcoming feedback on divergent students’ posts—highlighted in their positive contributions to discussion even if incorrect—seems to contribute to a learning environment which encourages the development of social competences that strengthen student participation.

## Complementary results

In the episode from l.40 to l.140, the results analyses within our two perspectives are particularly interesting and highlight different aspects of the mathematical discussion. At the beginning of this extract (Fig. 8), the teacher gives the corrective feedback related to divergent assumptions that emerged in the analysis from a perspective of inclusivity. In the first part of the graphical representation of this extract (Fig. 4) proposed in the mathematics education perspective, we observe that the interaction takes place at class level. In this interaction, we observed a change in focus: it is firstly pronounced, then attended to, and finally intended. Classroom interaction brings a progression in the discourse: we observe the interaction between the teacher and three students whose interventions, including gestures and verbal production both within text and upon text, make it possible to converge towards the idea that the growth of a person is not a linear process, and thus the original assumption cannot be considered completely satisfactory. As highlighted in the inclusion-oriented analysis, the discussion on this assumption ended with an explicit correction by the teacher of the initial assumption and evaluation of positive elements of the given answer (l.75–1.83). The fact that the divergent assumption was included in the Padlet gave the teacher the opportunity to analyse, discuss and correct this assumption, adopting the error as a learning opportunity for all the students without focusing on who proposed it. In her intervention, the teacher referred explicitly to the strategies included in the Padlet and then changed the interaction level of the discussion; this part of the dialogue is represented in green in the graphical representation. Another important turning point is from l.90 on, where a change of interaction level comes alongside the appearance of new mathematical elements (a conflict about the word ‘height’ and new measures such as the ‘proto-triangle’). This turning point was stimulated by an intervention of the teacher, who brought attention to the comments in the Padlet (red arrows and gestures, Fig. 4).

In fact, in this episode we observe many interactions between the teacher and students that are often accompanied by gestures; we observe different type of gestures (Gm - Ge - G). Gestures also act as support for verbal language, which is not always exhaustive or correct (e.g., in l.60 the gesture clarifies the use of the word ‘big’ instead of ‘high’). From the perspective of inclusive education, this action has been identified as a practice, activated and legitimated by the teacher, that values plural means of expression and integrates them in classroom communication.

## Discussion

The Complementary Accounts Methodology applied in analysis of our rich dataset gave us the opportunity to create a more in-depth portrayal of mathematical discussion. The experts involved in this research operated in sufficiently different fields to implement this approach, bringing complementary interpretations that reinforced each other. The richness of the data collected enabled the analysis of classroom mathematical discussions from different but coherent perspectives (Chan & Clarke, 2017).

The mathematics education analysis showed that use of the Padlet enlivens the discussion: the dynamics of the discussion were analysed on the basis of four levels of interaction, continuously intertwining. The analysis from the mathematics teaching perspective highlights two main general aspects of the mathematical discussion within the complex structure of the MHE, as follows:

1. MHE role in *mathematics learning*. From this standpoint, one can see learning “as the construction of a web of connections of mathematical ideas […]. These connections […] can be (re)constructed by students, not only through the scaffolding by a teacher but also by themselves through the use of digital environments” (Trouche & Drijvers, 2014, p. 195). The interactive flowchart illustrates precisely these specific features allowed by the MHE.
2. MHE role in *communication*. Within the communicational framework it does not “reduce to mere auxiliary means that come to provide expression to pre-existing, pre-formed thought. Rather, one thinks about them as part and parcel of the act of communication and thus of cognition” (Kieran et al., 2003, p. 29).

The mathematical discourse developed in the class touches upon different aspects of mathematical sense-making. On one hand, this means that many different mathematical facets are made palpable through a ‘melting pot’ of variety of meanings suggested by the different representations evoked and present in the MHE. On the other hand, as pointed out above, it is precisely this multiplicity of meanings that makes the mathematical content accessible to a wider audience in the classroom.

The results of the inclusion perspective showed the potential of the use of Padlet. It opens the door to an increased participation because of the coherent use of posts and comments activated by the teacher in the classroom discussion. The Padlet itself would not be enough if it were not coherently intertwined with the classroom conversation: it is the teacher who refers to some students’ posts when they do not speak during class conversation, legitimising a different type of participation. Moreover, the teacher promotes a positive culture of error and recognises a plurality of valid ways of expression and contributions to knowledge co-construction. In addition, the teacher promotes the development of students’ social competencies: she involves everyone in the discussion, motivates students to propose new issues in discussion and encourages the understanding of divergent assumptions.

The role of the teacher in orchestrating mathematical discussion emerges as fundamental also in analysis from the mathematics education perspective. As highlighted by the graphic representation of discussion analysis and by the statistics of Fig. 10, the teacher plays the role of mediator and conducts the discussion through verbal and gestural references which contribute to linking the four levels of interaction. Then, the Complementary Approach analysis shows how the ‘plural means of expressions integrated in classroom communication’ promoted by the Padlet, assume both an epistemological and a socio-pedagogical function, thanks to the crucial mediation of the teacher, who aptly takes advantage of the MHE to make the multiple means of expression available and comprehensible to students.

## Conclusions and further issues

Each different perspective highlighted elements in the dataset and, in particular, in the selected episode, which were useful in interpreting (from each specific point of view) the development of the classroom discussion, converging towards a mathematical interpretation of the situation and of the task with participation in the classroom discourse. These parallel analyses showed that mathematical discussion in the classroom is a complex (and sometimes chaotic) phenomenon wherein different factors interweave. A complementary approach is very fruitful for a global vision of this complex and dynamic evolution. On the other hand, if we look at certain passages from the discussion that have been highlighted by different perspectives, we see that there are local episodes that are crucial for this interweaving, and that it is precisely in these episodes that the role of the teacher is fundamental. These episodes appear as turning points and catalysts for the different variables, and the teacher acts as mediator in this process. In our case, mathematical, gestural, and verbal variables were actually interacting, and their roles were highlighted by the different theoretical perspectives. Resonances emerged in the analysis of the variables within the different perspectives.

We may conclude (RQ1) that the MHE supplied explicit elements to the mathematical discussion in the classroom, used by both the teacher and the students, and allowed an enriched discussion. It provided external inputs and enhanced a critical analysis of statements and argumentations. Indeed, the teacher or students can re-voice the texts in the Padlet in a reactive or proactive form, producing texts upon or against the Padlet posts. The teacher can also use the Padlet with a ‘Sherpa function’ to promote the discussion and manage the continual intertwining between the four levels of interactions.

Apart from mathematical facts, it appears that (RQ2) data highlighted the role of the MHE in promoting pupils’ participation in the mathematical discussion, for instance involving also students who generally choose not to speak during the class discussion. In the MHE, a crucial role is played by the means of expression students choose to adopt in order to take an active role in the discussion (write or comment a post on Padlet vs. pose or answer questions in the classroom discussion) and in the way the teacher legitimates these, emphasising each of their interventions as far as possible, and developing a positive attitude towards the error. One open issue is to investigate how these dynamics depend on the specific features of the class and the teacher’s style of teaching.

Finally (RQ3), the role of the teacher in orchestrating the discussion in an MHE is crucial from both perspectives: the teacher promotes the connection between a variety of voices, managing the dynamics of the different levels of interaction and promoting students’ participation. The Complementary results showed the importance of using plural means of expressions integrated in classroom discussion, in order to reach a co-construction of knowledge, and the MHE prompts students to participate in different ways. Students are encouraged to participate in the mathematical discussion, and the learning environment is recognised as positive and collaborative in both perspectives: indeed, there is a positive culture of errors, and divergent hypotheses are encouraged.

## Appendix

Transcript of the episode analyzed (lines 40–140): transcript in Italian (left) and its translation (right).

## Footnotes

1 The original activity can be consulted at http://www.scuolavalore.indire.it/nuove_risorse/la-foto/.
